# ﻿Morphological, acoustic and genetic identification of a reproducing population of the invasive African clawed frog *Xenopuslaevis* (Anura, Pipidae) recently discovered in Belgium

**DOI:** 10.3897/zookeys.1184.103702

**Published:** 2023-11-14

**Authors:** Olivier S. G. Pauwels, Jonathan Brecko, Dimitri Baeghe, Jeroen Venderickx, Ann Vanderheyden, Thierry Backeljau

**Affiliations:** 1 Scientific Heritage, Royal Belgian Institute of Natural Sciences, Rue Vautier 29, B-1000 Brussels, Belgium Scientific Heritage, Royal Belgian Institute of Natural Sciences Brussels Belgium; 2 Royal Museum for Central Africa, Leuvensesteenweg 13, B-3080 Tervuren, Belgium Royal Museum for Central Africa Tervuren Belgium; 3 Evolutionary Biology and Ecology (CP 160/12), Department of Organismic Biology, Faculty of Sciences, Université Libre de Bruxelles, Avenue F.D. Roosevelt 50, B-1050 Brussels, Belgium Université Libre de Bruxelles Brussels Belgium; 4 Operational Directorate Natural Environment, Royal Belgian Institute of Natural Sciences, Vautierstraat 29, B-1000 Brussels, Belgium Operational Directorate Natural Environment, Royal Belgian Institute of Natural Sciences Brussels Belgium; 5 Barcoding Facility for Organisms and Tissues of Policy Concern (BopCo), Royal Belgian Institute of Natural Sciences, Vautierstraat 29, B-1000 Brussels, Belgium Barcoding Facility for Organisms and Tissues of Policy Concern (BopCo), Royal Belgian Institute of Natural Sciences Brussels Belgium; 6 Evolutionary Ecology Group, University of Antwerp, Universiteitsplein 1, B-2610 Antwerp, Belgium University of Antwerp Antwerp Belgium

**Keywords:** Amphibians, France, freshwater biodiversity, invasive species, morphology, phylogeny, South Africa, systematics, taxonomy

## Abstract

Using external morphology of adults and tadpoles, osteology from high-resolution microcomputed tomography, vocalization analysis, and DNA sequence data, the identity of a reproducing Belgian population of invasive *Xenopus* at the current northernmost edge of the distribution of the genus in Europe was assessed. All data concur to an identification as Xenopus (Xenopus) laevis (Daudin, 1802). Genetically it is most closely related to populations of the Cape region in South Africa. No studies on the natural history of the Belgian *Xenopus* population and its impact on the local environment have been made to date.

## ﻿Introduction

The African clawed frog genus *Xenopus* Wagler, 1827 was recently revised by Evans et al. (2015) who described six new species and revalidated another, bringing the number of currently recognized species to 29, distributed among two subgenera. Their revision suggested that several populations might represent additional, undescribed species. Contrary to most species of *Xenopus* whose biology is poorly known, X. (Xenopus) laevis (Daudin, 1802) has been the subject of thousands of scientific and popular publications, because it is easy to breed in captivity and has been extensively used as an experimental model in biological and medical laboratories, and as a pet all over the world since the 1950s (Deuchar 1972; Cannatella and de Sá 1993; Gurdon and Hopwood 2000; Robert 2020; Fainsod and Moody 2022). Many individuals were released intentionally outside the species’ natural range or escaped from captivity, and the exceptional adaptability of this species, which originates from southern Africa, allowed it to establish reproductive populations in the wild in numerous countries in North and South America, in Asia, and in Europe (Peralta-García et al. 2014; Courant et al. 2017; Rödder et al. 2017; Mora et al. 2019; Wang et al. 2019). Within Europe, the northernmost populations were located in Wales and Lincolnshire in Great Britain, but they were recently extirpated, probably due to extreme weather conditions (Tinsley et al. 2015). The second northernmost populations within Europe were those of northern France, under study for two decades (Courant et al. 2017, 2018a, b, 2019), until the species was subsequently reported further north in a few Belgian localities along the French border (Gombeer et al. 2022; van Doorn et al. 2022).

External morphology is homogeneous among *Xenopus* species, and characters to differentiate them are often subtle (Evans et al. 2015). For instance, a reproductive population in Florida, first identified as *Xenopuslaevis* by Hill et al. (2017) based on external morphology, was later shown, with the help of DNA sequence data and high-resolution microcomputed tomography, to belong to X. (Silurana) tropicalis (Gray, 1864) (Goodman et al. 2021), a species with a different body size and physiology, and hence potentially different invasion impacts and spreading abilities. DNA sequence analyses by De Busschere et al. (2016) demonstrated that the invasive French populations of *Xenopuslaevis* involved two lineages, one from the southwestern Cape region, one from the northern area of South Africa (also see Furman et al. 2015). The expanding French populations of *Xenopuslaevis* at the edge of the species’ distribution have shown morphological changes facilitating high dispersal abilities, in particular in the relative length of the hind limbs in both sexes (Courant et al. 2019), as well as physiological changes allowing to survive cooler weather conditions (Araspin et al. 2020; Padilla et al. 2020).

Considering that the systematics of the genus *Xenopus* is still in progress, that some introduced populations might disappear without having been accurately characterized, that expanding populations experience rapid morphological and physiological adaptations, and that a proper taxonomic identification allows to better understand the natural history, propagation risks and potential environmental impacts of an introduced population, it is important to voucher and document this northernmost, Belgian population. We do so hereafter, with the help of external morphology, osteology using high-resolution microcomputed tomography, DNA sequence data, and acoustics.

## ﻿Materials and methods

**Figure 1.**
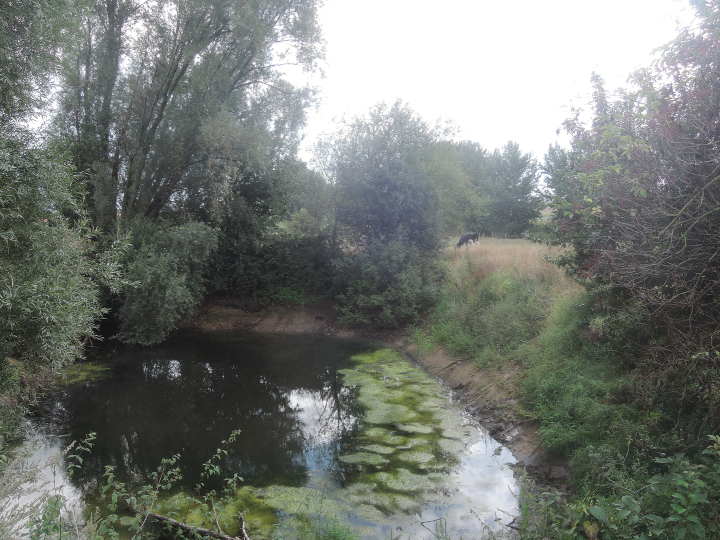
Pond in Comines-Warneton village, Hainaut Province, Wallonia, Belgium, where *Xenopuslaevis* were collected for the present study. Photograph by OSGP in September 2022.

Using baited funnel traps and scoop nets, we (DB, JB, OSGP, and AV) collected *Xenopus* individuals in the afternoons of 8 and 15 September 2022 in a pond (50°45'19.7"N, 2°53'8.9"E) in Comines-Warneton village, Hainaut Province, Walloon Region, Belgium. The pond is located in an agricultural area between a corn field and a cow pasture. It is not connected to a stream, but located at a dozen meters from another pond, itself at a dozen meters from the Douve stream, an affluent of the Lys (or Leie) River, a left-bank tributary of the Scheldt (Escaut) River. The pond, partly shadowed by *Salix* trees and a *Malus* apple tree, is largely invaded by filamentous algae (Fig. 1). At the time of our visits, the pond’s maximum depth was approximately one meter.

Among the individuals collected, 11 were kept alive in two aquariums for behavioral observations. All others, i.e., 19 subadult and adult specimens and 14 tadpoles, were preserved as vouchers, euthanized with 10% ethanol following the procedure of the Institutional Animal Care and Use Committee (**IACUC**), fixed in 90% ethanol, and subsequently transferred into 70% ethanol for permanent storage in the herpetological reference collection of the Royal Belgian Institute of Natural Sciences (**RBINS**), Brussels.

The following external measurements (adapted from Tinsley 1973) were taken on the freshly preserved voucher specimens with a Mitutoyo Absolute Digimatic digital caliper to the nearest 0.02 mm: Body width: measured at widest point of abdomen in adults (in tadpoles, maximum width behind eyes); Eye diameter: horizontal diameter of the eye (circumorbital plaques not included); 5^th^ toe length: length of outer ventral surface of 5^th^ toe, from the base of the metatarsal to the toe tip; 1^st^ finger length: distance between base (between 1^st^ and 2^nd^ fingers) and tip of 1^st^ finger; Head width: measured along a line through the bases of the subocular tentacles; Hind limb length: distance between vent and tip of 5^th^ toe (limb extended as straight as possible); Internarial distance: distance between inner margins of nostril-bordering flaps; Interocular distance: minimal distance between eyes (eyes not including circumorbital plaques); Lower forelimb length: distance between outer angle of elbow to tip of 1^st^ finger; Nostril diameter: measured across long axis of nostril (including the bordering flap and papilla in adults); Snout length: perpendicular distance from the tip of the snout to a line through the subocular tentacles; Snout-vent length (SVL): measured from the snout tip to the anterior point of the vent; Tentacle length: measured from anterior base to tip of subocular tentacle; Tibia length: medial measurement along dorsal surface of tibiofibula.

Measurements specific to tadpoles include: Barbel length: length of extended barbel, from posterior base to tip (measured on the right side of the head except if the right barbel is damaged); Body length: measured straight along body-tail axis from snout tip to a point above the posterior extremity of the vent tube; Dorsal fin height: maximum height of the dorsal fin; Snout-eye distance: minimum distance between eye and snout tip; Snout-nostril distance: minimum distance between nostril and snout tip; Tail height: maximum tail height at the level of the posterior extremity of the vent tube or beyond; Tail length: measured from a point on the axis snout tip to tail tip above the posterior extremity of the vent tube to the tip of the tail; Tail muscle height: maximum height of the tail muscle at the level of the posterior extremity of the vent tube or beyond; Tail muscle width: width of the tail muscle above the posterior extremity of the vent tube; Total length: measured straight from snout tip to tail tip along body-tail axis (= sum of Body length + Tail length); Ventral fin height: maximum height of the ventral fin. The description of the tadpoles is adapted from Channing et al. (2012), Tapley et al. (2015) and Zahn et al. (2022); we follow Gosner (1960) to define their development stages. Paired measurements were taken on the right side.

The number of lateral line plaques of adults was counted on the right side between the posterior extremity of the eye and the vent; plaques outside the main plaque line are not counted. Sex of adults and subadults was determined based on the presence of a protruding cloaca (generally obvious in dorsal view) and the absence of nuptial pad on forearms (females), or on the presence of nuptial pads (even if sometimes the pads are not very contrasted in color, they are perceptible to the touch) on the fore arms and absence of protruding cloaca (males).

MicroCT scans were made at the RBINS facilities. Adult specimens were digitized using an EasyTom 150 (RX Solutions, Chavanod, France) with an aluminum filter at 10–30 W, 110 kV, 5.5–12.5 frames/s, 1440 projections per rotation, and 16.66 or 18.25 isotropic voxelsize for head scans, and 32.35 μm or 32.89 μm isotropic voxelsize for full body scans. Segmentation of the scans was done using Dragonfly software, v. 4.1 for Windows (Object Research Systems (ORS) Inc, Montreal, Canada, 2020). Visualization of the resulting meshes was done using GOM Inspect 2018 (Carl Zeiss GOM Metrology GmbH, Braunschweig, Germany).

Our voucher specimens were morphologically compared with literature data (Tinsley 1973, 1995; Slater et al. 2009; Evans et al. 2011, 2015, 2019; Hill et al. 2017 - taking into account the misidentification of *Xenopustropicalis* as *X.laevis* in the latter reference; Porro and Richards 2017; Blackburn et al. 2019; Goodman et al. 2021; and references cited therein) and with preserved material housed in the collections of the RBINS, Brussels, and of the Royal Museum for Central Africa (RMCA), Tervuren, listed in the Appendix 1 (only a small part of the extensive *Xenopus* material housed in these two institutions is listed, according to the material we have personally revised).

Vocalizations of captive specimens were recorded with a Xiaomi Redmi Note 8T. The recorded vocalizations were imported in Raven Lite 2.0.4 (Ithaca, New York; The Cornell Lab of Ornithology). Within the software, the background noise was filtered. As the noise on the recording was continuously present on the entire recording, an adaptive filter was used to remove it. In the default settings of the adaptive filter, the broadband option was checked to remove a narrowband interference from a broadband signal. The filter order was set to 10 and the ALE (Adaptive Line Enhancer) delay to 1.

DNA-based species identification was done by extracting the DNA from six tadpole specimens using the Nucleospin Tissue Kit (Machery-Nagel), following the manufacturer’s protocols. Fragments of the mitochondrial genome were amplified; cytochrome oxidase I (COI) gene using the primer pair LCO1490 and HCO2198 (Folmer et al. 1994), cytochrome b (Cytb) gene using the primer pair CytbI and CytbII (Kessing et al. 1989) and a fragment of the large ribosomal subunit (16S) were amplified using the primer pair 16Sar and 16Sbr (Kessing et al. 1989).

PCR amplifications were performed in a total volume of 25 µl, containing 2 µl of DNA and 0.2 µM of each primer, and using 2× Qiagen® Multiplex PCR Kit with HotStarTaq® DNA polymerase with a final concentration of 3 mM MgCl_2_. For all gene fragments, the PCR profile was 15 min at 95 °C followed by 35 cycles of 45 s at 95 °C, 45 s at 53 °C, and 60 s at 72 °C, with a final extension step of 10 min at 72 °C. All PCR products were purified using the ExoSAP-IT protocol (ThermoFisher) and were sent for bidirectional sequencing to Macrogen (Amsterdam, The Netherlands). Generated sequences were trimmed, corrected, and assembled using Geneious® v. 10.0.4 (Biomatters Ltd.). A consensus sequence was generated for each specimen. The generated COI sequences were only used for species validation and compared against GenBank using Geneious® v. 10.0.4 (Biomatters Ltd.) BLAST algorithm.

To allow a direct comparison, all Cytb and 16S sequences used by De Busschere et al. (2016) were downloaded and aligned using ClustalW in Geneious® v. 10.0.4 (Biomatters Ltd.). The alignments were checked for stop codons and trimmed to retain the 280 bp of the Cytb region and the 540 bp of the 16S region.

Haplotypes were determined by cutting alignments to equal length (final alignment size Cytb: 273 bp; 16S 544 bp) generating a Haplotype data file in DnaSP v. 6 (Rozas et al. 2017). Haplotype names agree with De Busschere et al. (2016). A minimum spanning haplotype network was made using PopART (Bandelt et al. 1999). All generated DNA sequences were deposited in GenBank (accession numbers: COI, OQ318517: OQ318522, Cytb, OQ343503: OQ343508 and 16S, OQ318448: OQ318453).

## ﻿Results

### ﻿Adult external morphology

**Figure 2.**
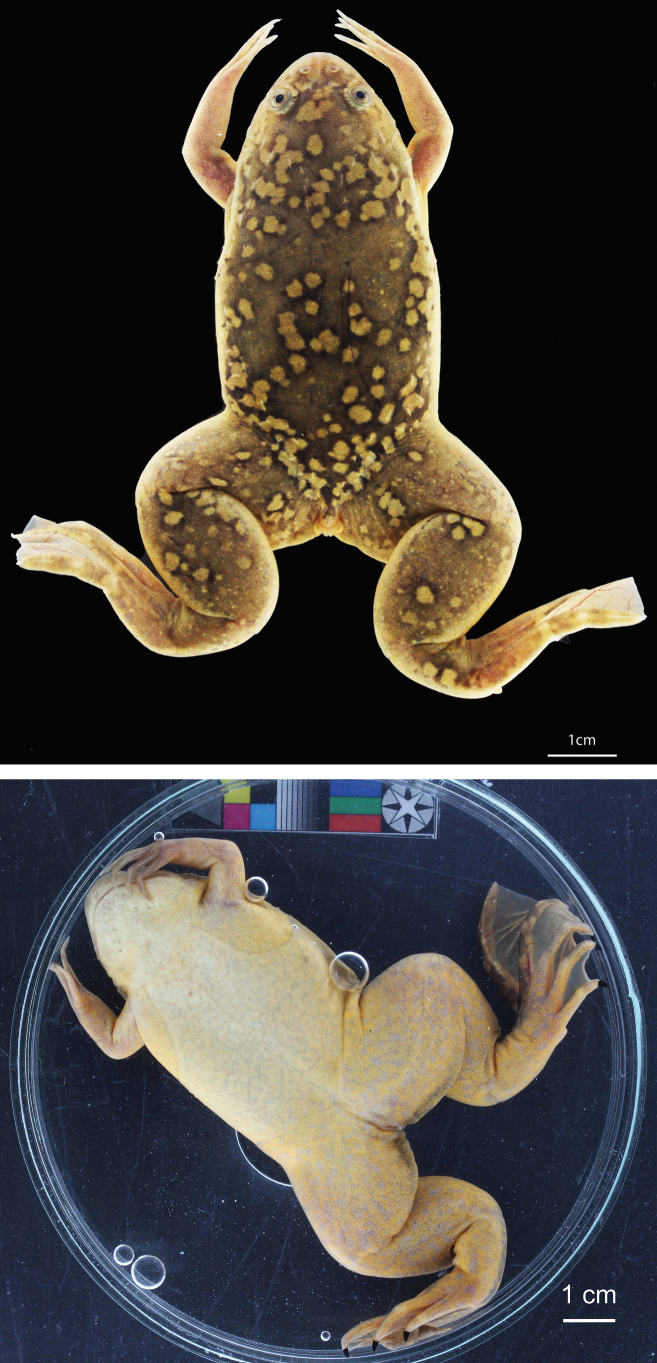
Adult female *Xenopuslaevis* from Comines-Warneton, Belgium, in dorsal (RBINS 18740) and ventral (RBINS 18744) views. Photographs by JV.

**Figure 3.**
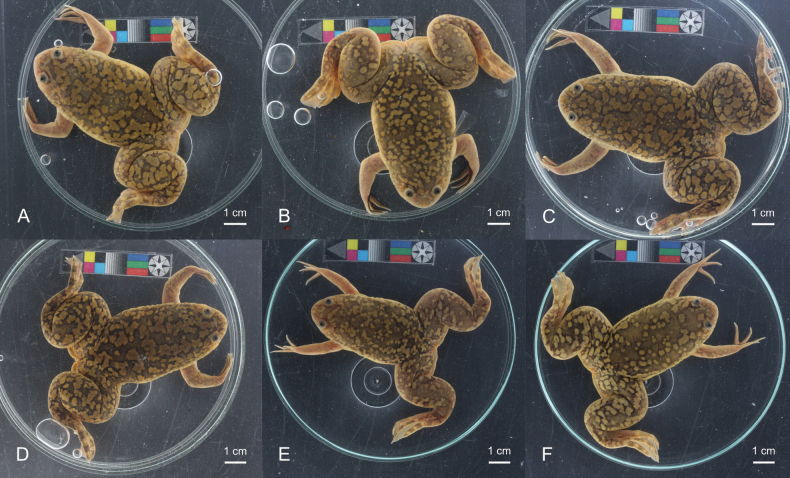
Dorsal pattern variation in adult male *Xenopuslaevis* individuals from Comines-Warneton, Belgium **A–F**RBINS 18732–18734, RBINS 18738, RBINS 18743 & RBINS 18745, respectively. Photographs by JV.

**Figure 4.**
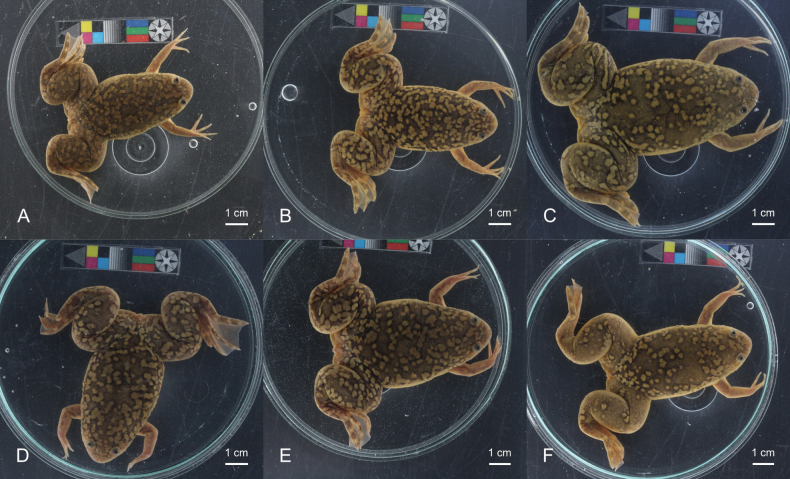
Dorsal pattern variation in adult female *Xenopuslaevis* individuals from Comines-Warneton, Belgium **A–F**RBINS 18731, RBINS 18735–18737 & RBINS 18739–18740, respectively. Photographs by JV.

External morphology data for freshly preserved adults and subadults are presented in Table 1. All measurements and counts could be taken on all specimens (9 males, 10 females). None of the specimens showed injuries or malformations, and all seemed to be well fed and in good health (Figs 2–4). Robust habitus. Body compressed dorsoventrally, oblong and ovoid in dorsal view. The largest individual, a female, shows a SVL of 74.84 mm. The SVL of the largest male is 69.38 mm. The maximum body width is 36.72 mm and is shown by the largest female. A distinctly larger individual was caught in the pond but escaped. The ratio body width / SVL varies between 0.42 and 0.51 (mean 0.46, Standard Deviation 0.02) in males, and between 0.45 and 0.50 (mean 0.48, SD 0.01) in females, showing no obvious sexual dimorphism. Forelimbs moderately robust. Hind limbs large and robust, with long feet. Ratio SVL / hind limb length between 0.72 and 0.84 (mean 0.78, SD 0.04).

**Table 1.** Morphometric (in mm) and meristic data for adult and subadult specimens of *Xenopuslaevis* from Comines-Warneton, Belgium. Dia = diameter; Dis = distance; L = length; W = width.

	RBINS 18731	RBINS 18732	RBINS 18733	RBINS 18734	RBINS 18735	RBINS 18736	RBINS 18737	RBINS 18738	RBINS 18739	RBINS 18740	RBINS 18741	RBINS 18742	RBINS 18743	RBINS 18744	RBINS 18745	RBINS 18746	RBINS 18747	RBINS 18748	RBINS 18749
Sex	F	M	M	M	F	F	F	M	F	F	F	F	M	F	M	M	F	M	M
SVL	50.81	64.60	69.38	65.07	55.10	70.32	59.09	64.94	66.89	62.21	74.84	67.11	56.43	69.46	58.69	59.17	55.37	55.72	53.74
Body W	23.17	29.83	29.01	30.95	26.21	31.51	29.50	28.52	32.32	28.67	36.72	32.19	25.49	34.36	29.84	28.02	25.85	26.43	23.52
Head W	11.38	15.18	15.29	14.73	12.00	15.59	14.11	14.18	14.63	13.49	16.51	15.08	12.88	14.73	14.28	13.65	12.30	12.94	12.42
Snout L	4.87	5.37	6.06	6.16	4.48	6.90	5.68	5.30	6.16	5.98	6.78	6.90	5.24	6.43	5.81	5.92	5.27	5.35	5.38
Eye Dia	3.27	3.86	3.50	3.37	3.37	3.68	3.42	3.66	3.59	3.23	3.65	3.82	3.08	3.68	2.95	3.46	2.93	3.30	3.28
Interocular Dis	7.03	8.89	9.14	9.10	8.49	10.13	8.39	8.90	8.85	9.20	9.96	9.51	7.99	9.65	8.31	9.02	7.80	8.23	7.65
Nostril Dia	1.17	1.66	1.85	1.53	1.66	1.67	1.54	1.70	1.40	1.69	1.93	1.90	1.39	1.82	1.69	1.81	1.37	1.49	1.39
Internarial Dis	2.01	2.55	2.38	2.64	2.10	2.93	2.59	2.61	2.69	2.28	2.70	2.41	1.81	2.48	2.37	2.38	2.10	2.40	1.96
Tentacle L	0.37	0.56	0.47	0.48	0.42	0.71	0.73	0.40	0.57	0.49	0.71	0.80	0.48	0.50	0.63	0.52	0.45	0.73	0.50
Lower forelimb L	18.49	28.42	28.26	28.40	20.59	26.46	22.02	27.20	23.81	22.47	27.89	24.95	21.94	24.83	24.31	26.42	19.06	23.70	20.60
1^st^ finger L	7.25	10.85	10.88	11.14	8.77	11.11	9.50	10.92	9.86	8.32	11.79	10.42	8.52	9.73	9.60	10.74	7.90	9.47	8.43
Hind limb L	61.20	78.99	85.43	89.78	74.06	89.60	74.73	89.80	85.01	80.34	95.20	85.52	75.05	87.33	71.23	80.39	65.91	75.92	68.84
Tibia L	22.37	28.75	29.02	30.15	23.78	29.38	24.71	28.44	27.29	25.63	30.83	30.29	24.90	29.37	26.34	27.08	22.70	25.33	23.41
5^th^ toe L	23.90	30.92	29.26	29.54	24.95	29.03	27.50	32.62	30.12	28.57	33.75	30.64	25.40	30.04	29.39	30.01	24.72	27.30	26.50
Lateral line plaques	27	23	27	24	23	28	27	25	28	30	27	26	24	28	24	23	27	25	24

Pupil round. Lower eyelid covering more than a third to half of the eye. Snout rounded in dorsal view, projecting distinctly beyond the lower jaw in lateral and ventral views. Nares ellipsoid, directed dorsally, with a small skin sheet projecting from their margin, and with each a nubbin on their lateral external extremity. Internarial region flat. Canthus rostralis not marked, flat. The ratio snout length / head width varies between 0.35 and 0.46 (mean 0.41, SD 0.03). Subocular tentacle not prominent, sometimes barely visible in life. The ratio tentacle length / eye diameter varies between 0.11 and 0.22 (mean 0.16, SD 0.03). Eyes moderate, the ratio eye diameter / interocular distance varies between 0.35 and 0.47 (mean 0.39, SD 0.03). Each eye encircled by lateral-line plaques on a raised ring of skin. Ratio nostril diameter / internarial distance between 0.52 and 0.79 (mean 0.68, SD 0.08). No visible tympanic annulus. Tongue absent. Choanae posteroventrally directed, large, oval. Mouth floor wrinkled and covered with flat pustules. No vomerine teeth. Loreal region flat to slightly concave.

Skin smooth, lacking asperities; no spicules on dorsal surfaces. Lateral line plaques present, stitch-like. A curved alignment of 23–30 plaques (mean 25.8, SD 2.02) on an irregular line between eye and cloaca. Other plaques are also irregularly distributed on the snout, the lateral and posterodorsal surfaces of the head, anterior part of dorsum, on the chin and throat, and on the flanks. Protruding cloacal lobes in females; cloacal lobes not fused ventrally.

Four fingers, very elongate and tapering to a pointed extremity, lacking webbing. In females, ventral surface of fingers covered with small conical spicules with a black point. In males, the spicules are much more developed, more numerous, and cover the ventral surfaces of fingers and hands as well as the internal surface of the arms, to form blackish nuptial pads whose rugosity is easily perceptible to the touch. Relative lengths of fingers: II > I ≥ III > IV. Ratio 1^st^ finger length / lower forelimb length between 0.37 and 0.43 (mean 0.40, SD 0.02). Five long toes; 1^st^, 2^nd^, and 3^rd^ with a keratinous claw. Feet fully webbed, till the base of the claws on the 1^st^ to 3^rd^ toes, till the extremity on 4^th^ and 5^th^ toes. Relative lengths of toes: IV > V > III > II > I. Ratio tibia length / 5^th^ toe length between 0.87 and 1.02 (mean 0.94, SD 0.04). Prehallux prominent but without a claw. Dermal ridge extending along the 1^st^ toe from the prehallux. No subarticular tubercles on hands and feet.

In life, the dorsal and lateral surfaces of the head, the dorsum and upper part of the flanks, the dorsal surfaces of arms and fingers, and of legs and toes of the adults show an irregular marbled pattern with rounded to elongate olive patches surrounded by darker olive-brown; these colors darken in preservative. The webbing of the feet is translucent olive with contrasting brown blood vessels. The lateral line’s stitches appear slightly lighter than the background color and are easily visible on live specimens. In living animals the iris is golden olive-brown, turning to blackish brown in preservative. The pupil is black in living animals and turns to white in preservative. The variation in the dorsal color pattern easily allows to individually recognize each specimen (a character facilitating the use of this species in laboratory studies, see Delpire et al. 2011, as well as in field studies). The underside of the head is whitish to pale beige, darkening in preservative, especially on the chin. The chest and belly are pale beige, of the same color as the underside of the head. The undersides of the arms, legs and feet are pale yellow. The ventral surfaces are immaculate or show some scattered blackish dots. Some individuals are dorsally and ventrally darker than others. The claws are black.

### ﻿Adult osteology

**Figure 5.**
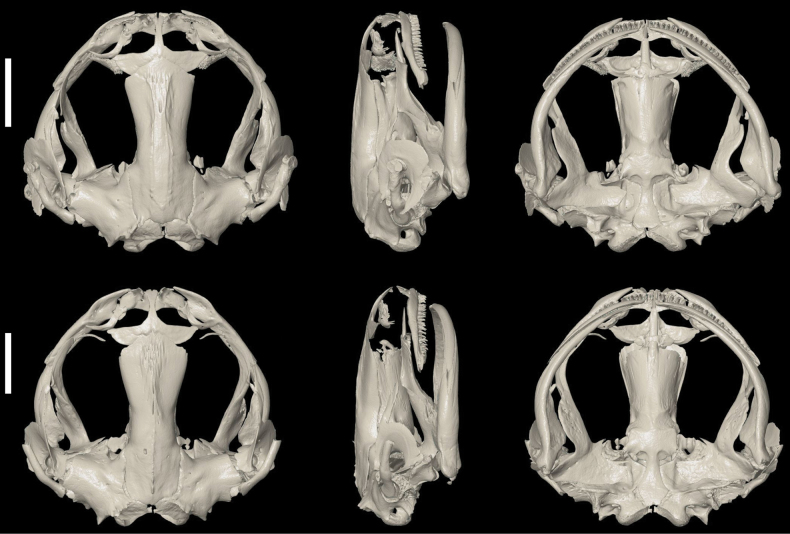
X-ray computed tomogram of the skull of adult *Xenopuslaevis* individuals from Comines-Warneton, Belgium in dorsal view, right profile and ventral view. Top = RBINS 18733 (male), bottom = RBINS 18736 (female). Scale bars: 5 mm. Scan by JB.

**Figure 6.**
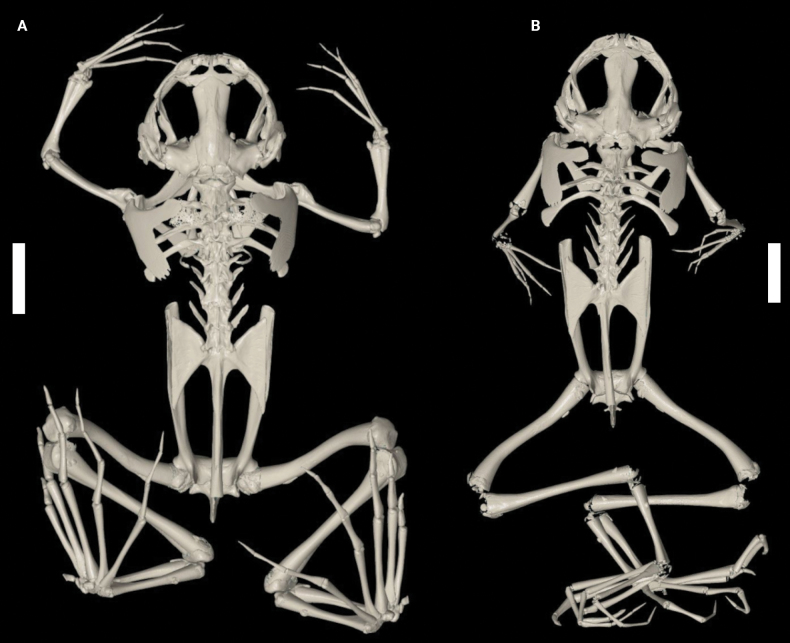
X-ray computed tomogram of the full skeleton of adult *Xenopuslaevis* (**A** male RBINS 18733 **B** female RBINS 18736) from Comines-Warneton, Belgium, in dorsal view. Scale bars: 1 cm.

The segmented skulls of RBINS 18733 (male) and RBINS 18736 (female) are shown in Fig. 5 in dorsal, lateral, and ventral views. The dorsal and lateral views of the skulls clearly show the elongate septomaxillae, the funnel-shaped squamosal, and the flattening of the posterior and medial rami of the pterygoid, which are unique for pipids (Porro and Richards 2017). The dorsal and ventral views of the skulls show that the nasals are fused, and the ventral view that a vomer is present. These two characters exclude the subgenus Silurana Gray, 1864 and unambiguously allocate these specimens to the subgenus Xenopus (Evans et al. 2015; Goodman et al. 2021). Premaxillary and maxillary teeth are present. Vomerine teeth are absent; within the genus *Xenopus*, they are known only in *X.fischbergi* Evans, Carter, Greenbaum, Gvoždík, Kelley, McLaughlin, Pauwels, Portik, Stanley, Tinsley, Tobias & Blackburn, 2015, *X.fraseri* Boulenger, 1905, and *X.muelleri* (Peters, 1844) (Evans et al. 2015, 2019; Porro and Richards 2017). The complete skeletons of the same two specimens are illustrated in Fig. 6, additionally showing that the 1^st^ two presacral vertebrae are clearly distinct, and not fused as in *Silurana*. The sacrum and urostyle are fused. A close examination of the parasphenoid reveals that no lateral alae are present. Typically, in pipids the parasphenoid has a cultriform process and a wide corpus, lacking posterolateral extensions, while other frogs generally have a tridiate parasphenoid possessing posterolateral extensions and an anterior cultriform process (Cannatella and Trueb 1988).

### ﻿Tadpole morphology

**Figure 7.**
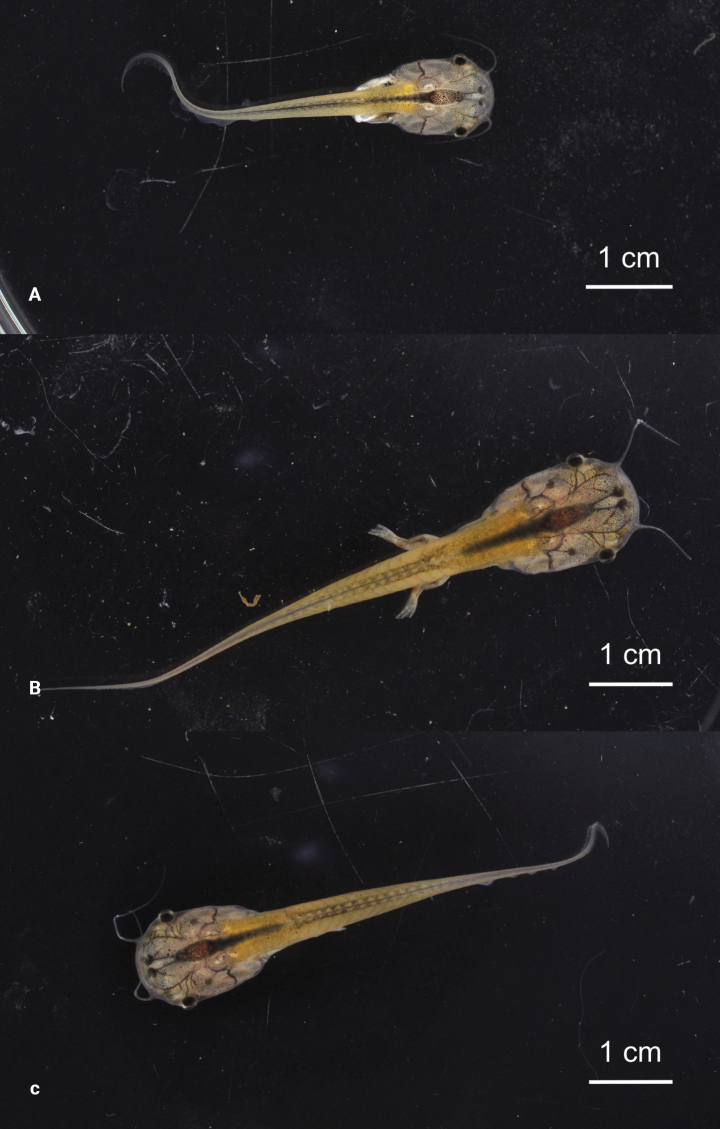
Tadpoles of *Xenopuslaevis* from Comines-Warneton, Belgium, in dorsal view **A–C**RBINS 18723–18725, respectively at Gosner’s stages 36, 40, and 40. Photographs by JV.

Dorsal views of three of the vouchered tadpoles are presented in Fig. 7. External morphology data for freshly preserved tadpoles are presented in Table 2. All tadpoles seemed healthy and did not show any external malformation. The levels of development of the six tadpoles collected on 8 September correspond to Gosner’s (1960) stages 31–37. The eight tadpoles collected a week later were all at stage 40, except one at stage 36.

**Table 2.** Morphometric data (in mm) for tadpoles of *Xenopuslaevis* from Comines-Warneton, Belgium. Dia = diameter; Dis = distance; H = height; L = length; W = width. NA = not available (damaged). * = tail tip cut for genetic analysis.

	RBINS 18717	RBINS 18718	RBINS 18719	RBINS 18720	RBINS 18721	RBINS 18722	RBINS 18723	RBINS 18724	RBINS 18725	RBINS 18726	RBINS 18727	RBINS 18728	RBINS 18729	RBINS 18730
Collecting date d/m/y	8/9/2022	8/9/2022	8/9/2022	8/9/2022	8/9/2022	8/9/2022	15/9/2022	15/9/2022	15/9/2022	15/9/2022	15/9/2022	15/9/2022	15/9/2022	15/9/2022
Gosner stage	36	37	36	33	37	31	36	40	40	40	40	40	40	40
Total L	>34.22*	>34.28*	>40.17*	>25.3*	>45.83*	>28.83*	65.09	70.84	61.07	57.15	55.38	53.04	61.86	65.24
Body L	20.91	22.30	22.94	18.10	23.86	17.23	18.03	27.19	23.25	24.70	22.09	20.89	23.61	25.19
Body W	8.45	9.39	10.70	6.50	8.47	6.43	6.67	9.58	8.73	8.30	7.46	5.67	6.47	8.43
Tail L	>13.31*	>11.98*	>17.23*	>7.20*	>21.97*	>11.60*	28.76	43.65	37.82	32.45	33.29	32.15	38.25	40.05
Tail muscle H	5.03	5.08	5.20	4.25	5.57	3.54	3.03	5.24	4.88	4.91	3.53	3.44	4.02	4.61
Tail muscle W	2.32	2.78	2.33	2.02	2.54	2.20	1.51	2.38	2.19	1.91	1.53	1.51	1.76	1.75
Tail H	6.16	6.73	6.90	NA	7.42	3.98	5.40	7.94	9.02	6.41	5.96	6.05	6.75	7.81
Dorsal fin H	0.46	0.50	0.80	NA	0.57	0.52	0.68	1.43	1.04	1.25	1.03	1.02	1.27	1.28
Ventral fin H	2.50	2.55	2.49	NA	2.11	1.21	2.37	3.17	3.70	2.94	2.60	2.48	2.36	3.20
Eye Dia	1.75	1.61	1.77	1.54	1.65	1.15	1.54	1.80	1.70	1.76	1.54	1.45	1.78	1.77
Interocular Dis	4.27	5.67	6.20	5.71	6.70	3.12	5.26	9.48	7.86	8.05	7.46	6.07	7.14	9.29
Nostril Dia	0.63	0.59	0.80	0.65	0.71	0.48	0.44	0.67	0.62	0.73	0.70	0.64	0.71	0.74
Internarial Dis	1.07	1.03	1.10	0.94	1.02	0.60	0.93	1.07	1.06	1.11	0.98	0.96	1.08	1.05
Snout-eye Dis	5.42	5.30	5.10	4.85	5.15	3.64	4.41	7.08	6.27	5.90	5.70	5.05	5.95	6.20
Snout-nostril Dis	2.19	1.74	1.58	1.52	1.99	1.21	1.50	2.14	2.21	1.80	1.83	1.45	1.78	1.84
Barbel L	8.25	10.83	10.90	7.58	10.52	9.44	7.55	12.31	13.01	12.07	10.77	8.71	12.95	15.74

The largest preserved tadpole at Gosner’s (1960) stage 40 (RBINS 18724) can be described as follows: Body oval in dorsal view. Body width 9.58 mm. Total length 70.84 mm (body length 27.19 mm, tail length 43.65 mm). Ratio tail length / total length 0.62 (mean ratio in all vouchered tadpoles at Gosner’s (1960) stage 40 0.61, SD 0.02). Dorsolaterally flattened head. Mouth terminal and slit-like. No keratinized mouthparts. A single barbel on each side, originating from the lateral extremities of the mouth (barbels present on both sides in all our vouchered tadpoles, at all stages of our sample). Barbel length 12.31 mm. Ratio barbel length / body length 0.45 (mean ratio in all vouchered tadpoles at Gosner’s stage 40 0.51, SD 0.06). Eyes in lateral position. Eye diameter 1.80 mm. Snout-eye distance 7.08 mm. Interorbital distance 9.48 mm. Nares transversely elliptical and parasagittal, located closer to the snout than to the eye. Narial diameter 0.67 mm. Internarial distance 1.07 mm. Snout-nostril distance 2.14 mm. Vent tube medial. Tail muscle height 5.24 mm. Low dorsal fin, originating at tail-body junction. Maximum dorsal fin height 1.43 mm. Ventral fin distinctly higher than dorsal fin. Ventral fin originating at mid-abdomen, increasing in height posteriorly till the opening of the vent tube, then slightly diminishing in height, and increasing again to reach its maximum height (3.17 mm) at approximately mid-length of tail, then progressively tapering towards the end of the tail, which terminates as a flagellum. Maximum tail height 7.94 mm. Tail muscle width 2.38 mm. All five toes well distinct, fully webbed. Forelimbs visible through the transparent skin.

In preservative, head and body whitish, translucent, with numerous scattered, blackish melanophores. The pupil, black in life, turns to white in preservative. Barbels whitish, translucent. Tail muscle yellowish. Tail fins transparent.

### ﻿Vocalizations

**Figure 8.**
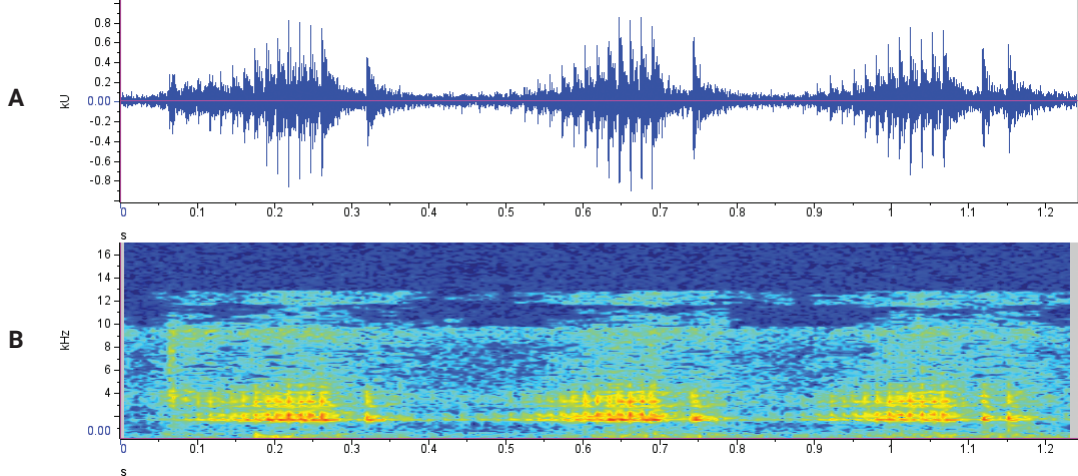
Oscillographs (**A**) and spectrographs (**B**) for three trill bouts recorded at 00:15 am from captive *Xenopuslaevis* caught in Comines-Warneton, Belgium (water temperature 22 °C). Images by JB.

In the laboratory four males and two females were housed together in a large tank. At night (00:15 am, 18 September 2022), in complete darkness, we recorded a short series of calls. This happened just after the frogs were startled by movement in the laboratory. The oscillographs (Fig. 8A) and spectrographs (Fig. 8B) show three trill bouts of a larger train of sound pulses. These trill bouts are 232 ± 14 ms (Mean ± SD) long. The recorded calls fit within the classification of chirping vocalizations (Tobias et al. 2004; Leininger and Kelley 2015). These calls are typically produced within male-male interactions. Such interactions might be during clasping and the trills can be produced by both clasping and clasped males as studies of Tobias et al. (2004) on *Xenopuslaevis* showed. However, no clasping was witnessed while these calls were produced.

### ﻿mtDNA

**Figure 9.**
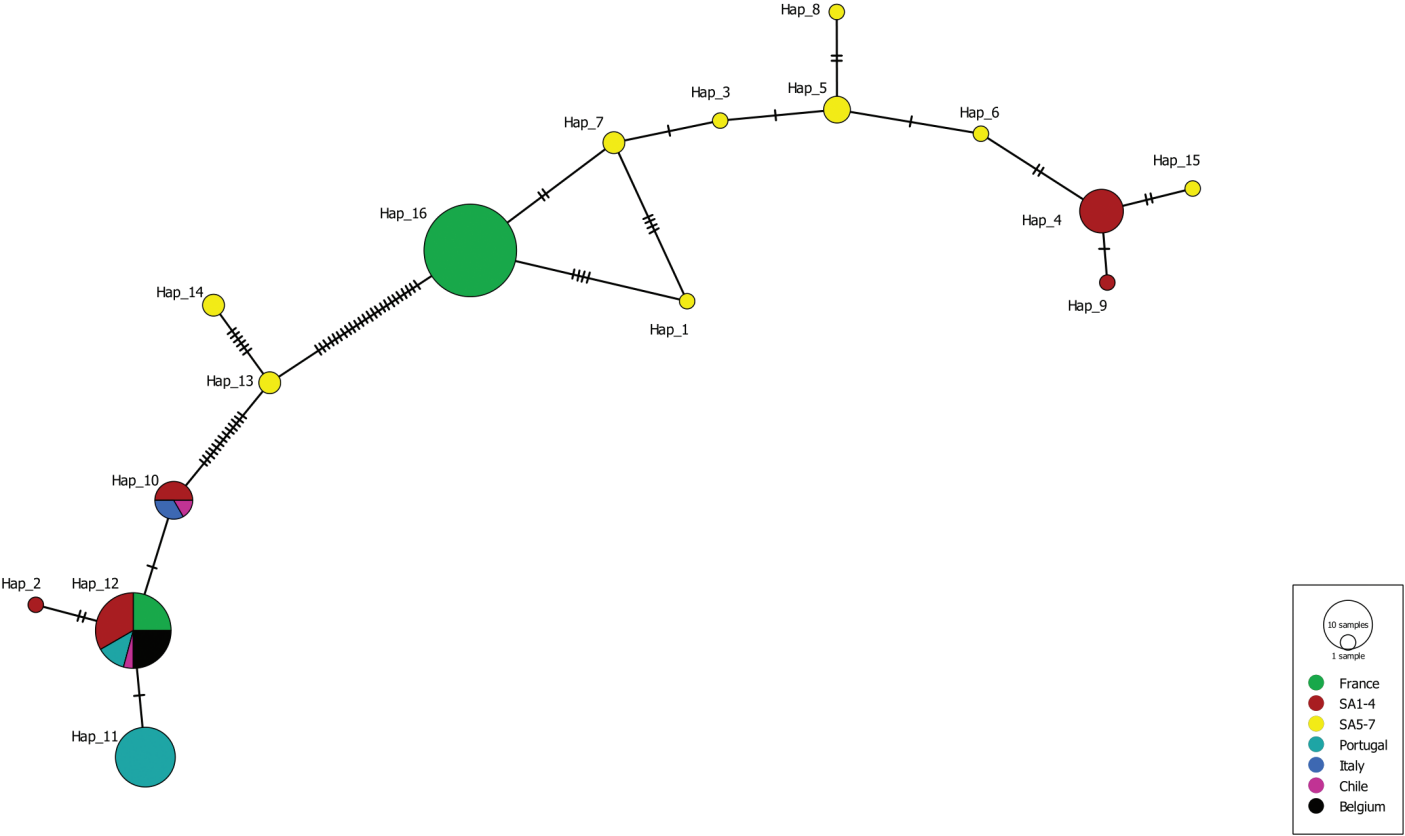
Minimum Spanning Haplotype network of Cytb sequences of *Xenopuslaevis*, newly generated sequences are indicated as ‘‘Belgium”. The sizes of the circles are proportional to haplotype frequencies. Colors refer to native (South Africa) and invaded regions (see legend). Numbers of mutations are marked by stripes on the connecting branches. Abbreviations and haplotype names follow De Busschere et al. (2016): Belgium = Comines-Warneton, Italy = Sicily, SA1 = southwestern Cape, SA2 = Cape, Hoekwil & Tsitsikamma region, SA3 = Cape, Laingsburg, SA4 = Cape, Beaufort West, SA5 = northern South Africa (Kimberley, Victoria West, Potchefstroom), SA6 = Cape, Rooikrantz Dam, SA7 = Nieuwoudtville.

**Figure 10.**
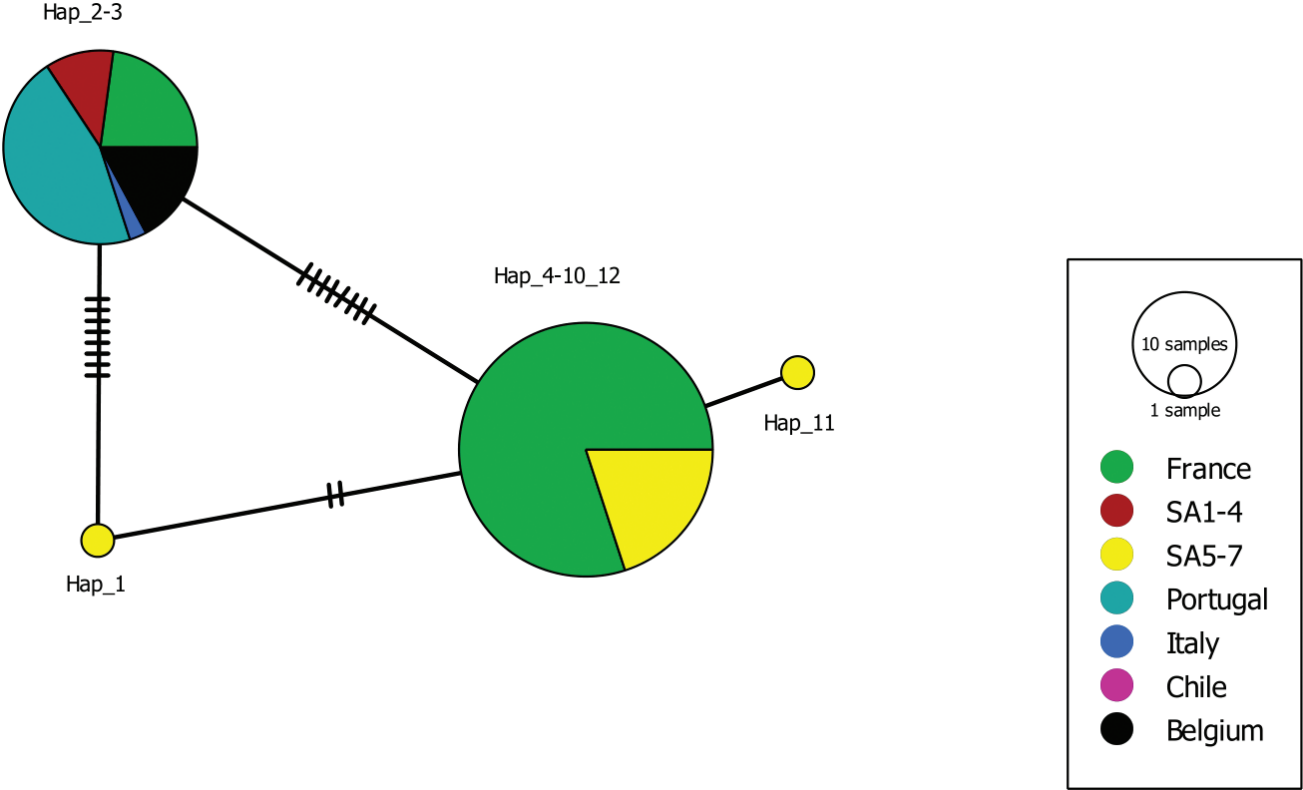
Minimum Spanning Haplotype network of 16S sequences of *Xenopuslaevis*. The sizes of the circles are proportional to haplotype frequencies. Colors refer to native (South Africa) and invaded regions (see legend). Numbers of mutations are marked by stripes on the connecting branches. Newly generated sequences are indicated as ‘‘Belgium’’. Abbreviations and haplotype names follow De Busschere et al. (2016), see Fig. 9.

All generated COI sequences of *Xenopuslaevis* from Comines-Warneton (Belgium) were identical to several GenBank sequences of *X.laevis* from France (Fr), Portugal (Pt), and South Africa (SA1, southwestern Cape; SA4, Beaufort West; SA7, Niewoudtville) (Fr: OP108330; Pt: OP108328; SA1: OP108302; SA4: OP108304; SA7: OP108299; Schoeman et al. 2022). The Cytb alignment involved 16 unique haplotypes of *Xenopuslaevis* and the 16S alignment involved 15 unique haplotypes (De Busschere et al. 2016). The Cytb sequences of the *Xenopuslaevis* specimens from Comines-Warneton were all identical to “Hap_12” (Fig. 9) observed in specimens from Chile, France, Portugal, and South Africa (De Busschere et al. 2016). Because the 16S fragment of the specimens from Comines-Warneton was shorter (± 370 bp), only four haplotypes could be recovered (Fig. 10). For this shorter 16S fragment “Hap_2” and “Hap_3” (sensu De Busschere et al. 2016) could not be distinguished and was found to be identical to the generated 16S sequences of *Xenopuslaevis* in Comines-Warneton. 16S Haplotypes “Hap_2” and “Hap_3” were observed in specimens from France, Italy, Portugal, and South Africa (De Busschere et al. 2016).

## ﻿Discussion

The combination of cloacal lobes not ventrally fused, absence of a claw on prehallux, absence of spicules on dorsum, presence of a dermal ridge along the 1^st^ toe from the prehallux, relatively long feet, relatively large eyes, more than a third of the eye covered by the lower eyelid, fused nasal bones, and the presence of vomer bones in the palate unambiguously shows that the *Xenopus* population from Comines-Warneton belongs to the subgenus Xenopus (see diagnostic characters in Evans et al. 2015), excluding the subgenus Silurana and the four species it currently contains (X. (S.) calcaratus Peters, 1875, X. (S.) epitropicalis Fischberg, Colombelli, & Picard, 1982, X. (S.) mellotropicalis Evans, Carter, Greenbaum, Gvoždík, Kelley, McLaughlin, Pauwels, Portik, Stanley, Tinsley, Tobias & Blackburn, 2015, and X. (S.) tropicalis (Gray, 1864)). Within the subgenus Xenopus, their dense marbled dorsal pattern readily separates the clawed frogs of Comines-Warneton from all species but *X.borealis* Parker, 1936, *X.laevis*, *X.petersii* Barboza du Bocage, 1895, *X.poweri* Hewitt, 1927 and *X.victorianus* Ahl, 1924, the latter three species being closely related to *X.laevis* but much smaller. The combination of the large size of adult males (SVL to at least 69.38 mm) and the absence of vomerine teeth distinguishes the clawed frogs of Comines-Warneton from all species of the subgenera *Silurana* and *Xenopus* except *X.laevis* (Evans et al. 2015, 2019; Porro and Richards 2017).

The tadpole morphology of many *Xenopus* species is unknown or fragmentary, often limited to a few Gosner’s (1960) stages. Still, a comparison among the known tadpoles shows strongly similar morphologies with few obvious differences (Channing et al. 2012; Evans et al. 2015; Tapley et al. 2015). However, the size, the low density, and the distribution of the melanophores on the dorsum and tail, as well as the relative shortness of the barbels agree with an identification as *Xenopuslaevis* (Vigny 1979).

**Figure 11.**
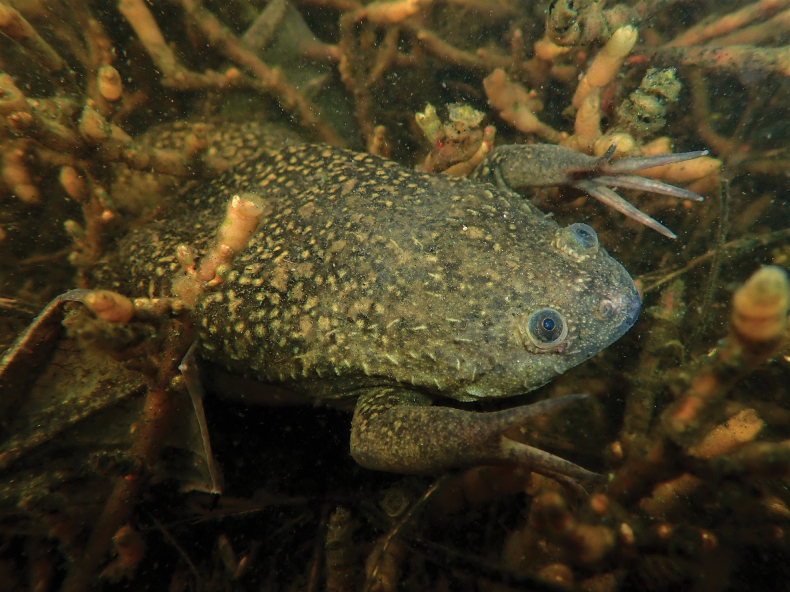
Adult *Xenopuslaevis* in a wetland (34°7'0.2"S, 18°22'39.3"E) at the Lake Michelle eco estate, Noordhoek, Western Cape, South Africa. Photograph by M. Burger.

De Busschere et al. (2016) identified two phylogeographic lineages of South African origin invading France, one originating from northern South Africa and Rooikrantz Dam (SA5–SA6, referred to as the northern lineage), the other originating from the southwestern Cape (SA1–SA2). Despite the fact that 86% of the French specimens in the study of De Busschere et al. (2016) belonged to the northern lineage, sequences from the specimens from Comines-Warneton were most similar to sequences from *Xenopuslaevis* from the Cape region (SA1–4, Figs 9–11).

**Figure 12.**
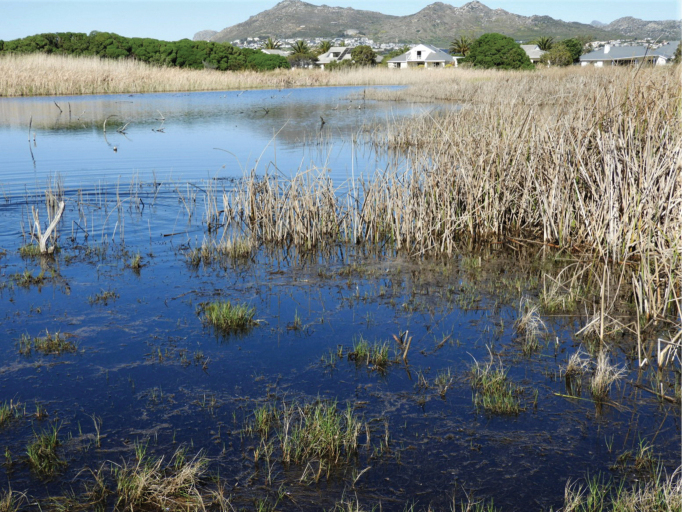
Wetland (34°7'0.2"S, 18°22'39.3"E) at the Lake Michelle eco estate, Noordhoek, Western Cape, South Africa, home to *Xenopuslaevis*. Photograph by M. Burger.

Including our voucher series, plus the 11 specimens kept alive for behavioral observations and several specimens that escaped in situ, the sex ratio is approximately 1:1. In the natural environment of *Xenopuslaevis* in South Africa (Fig. 12), females are often slightly more numerous than males but both sexes are sometimes found in equal proportions (Du Preez et al. 2005). However, it is known that males and females may respond differently to baited traps (Lobos and Measey 2002).

While collecting *Xenopus*, two other amphibian species were observed in the pond of Comines-Warneton during our first visit: four juvenile *Lissotritonvulgaris* (Linnaeus, 1758) (Salamandridae) and two legged tadpoles and one just-metamorphosed individual of *Pelophylax* sp. (Ranidae). We observed no fishes in the pond, but we did observe high densities of insect larvae, including Odonata (Aeshnidae: *Anax* sp.; Coenagrionidae: *Ischnura* sp.; Libellulidae: *Orthetrum* sp., etc.), and aquatic Coleoptera (Dytiscidae: *Agabusnebulosus* (Forster, 1771), *Hyphydrusovatus* (Linnaeus, 1761); Hygrobiidae: *Hygrobiahermanni* (Fabricius, 1775), etc.) and aquatic Heteroptera (Corixidae: *Corixapunctata* (Illiger, 1807); Notonectidae: *Notonectaglauca* (Linnaeus, 1758), etc.). During our visit to the pond on 10 October 2022, numerous adult *Xenopuslaevis* were still actively going to the surface to breath, but not a single one was caught in our baited traps. This may indicate that, with the cooler weather of October and in preparation for the winter, they stop feeding or decrease their foraging activities.

**Figure 13.**
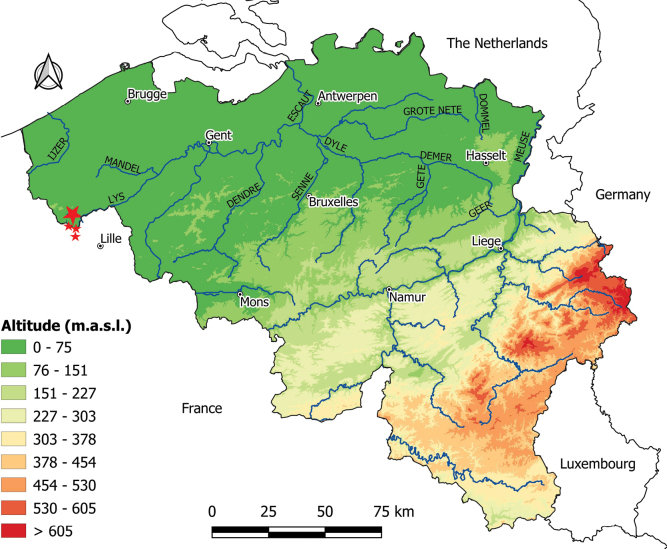
Map of Belgium showing the location of the pond in Comines-Warneton (largest, northernmost, red star), Hainaut Province, Wallonia. The three other red stars, smaller, show, from north to south, the locations of Le Bizet, Rau d’Esseu, and La Chapelle d’Armentières, respectively. Map by A. Tovar and JV.

**Figure 14.**
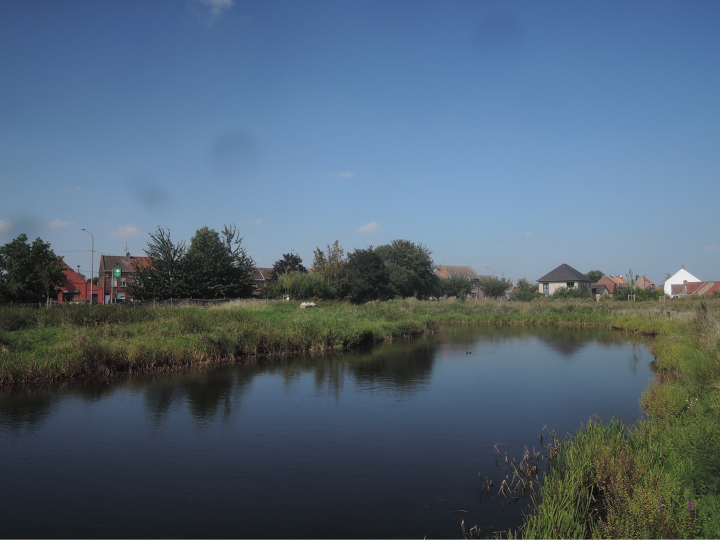
Pond in La Chapelle d’Armentières, Nord Department, France, housing the northernmost French population of *Xenopuslaevis*. Photograph by OSGP in September 2021.

It is difficult to assess when the pond in Comines-Warneton was first colonized by *Xenopuslaevis*. The first observations of *Xenopus* in Belgium were made in 2006 in a pond (50°42'30.3"N, 2°52'43.3"E) in Le Bizet, and in a river directly connected to the Lys, the Rau d’Esseu (50°41'56.3"N, 2°54'28.0"E; van Doorn et al. 2022), at just 5.5 km S and 7 km S-SE, respectively, from the pond of Comines-Warneton (Fig. 13). The geographically closest confirmed established population of *Xenopuslaevis* lives in a pond (50°40'13.4"N, 2°54'24.6"E; Fig. 14) in La Chapelle d’Armentières, northern France, at less than 10 km south of the pond in Comines-Warneton (Fig. 13). Given the relative isolation of the Belgian pond studied here, the abundance of *Xenopuslaevis* in the pond, and the presence of tadpoles as well as subadults and large adults, it is certain that the pond has housed *X.laevis* for several years, and most probably a number of neighboring streams and ponds are also supporting them. The species is so secretive that the Western France population was discovered two decades after its introduction (Lorrain-Soligon and Secondi 2022). According to our discussions with local farmers, there is no indication that the propagation of *Xenopuslaevis* from the closest locality in France was human-mediated. This implies that *Xenopuslaevis* may have used the Lys and several of its affluents, at least the Douve and the Rau d’Esseu, to travel from France.

Studies on sites in western France where *Xenopuslaevis* is established suggest a negative impact on the native amphibian fauna and on the aquatic macroinvertebrate communities (Courant et al. 2018a, b). No studies on the natural history and the potential environmental impact of Belgian *Xenopuslaevis*, at the northern edge of the genus’ distribution, have been made so far. The pond in Comines-Warneton, inhabited by at least two other amphibian species, is an ideal site for such studies.

## ﻿Additional information

### Author ORCIDs

Olivier S. G. Pauwels https://orcid.org/0000-0002-3265-5496

Jonathan Brecko https://orcid.org/0000-0002-2969-4566

Dimitri Baeghe https://orcid.org/0000-0003-2522-5954

Jeroen Venderickx https://orcid.org/0000-0002-4487-2009

Ann Vanderheyden https://orcid.org/0000-0003-2823-0855

Thierry Backeljau https://orcid.org/0000-0002-9057-9727

### Data availability

All of the data that support the findings of this study are available in the main text.

## ﻿Appendix 1

Comparative material examined. The number of specimens is specified when it is above one. “DRC” = Democratic Republic of the Congo; “Prov.” = Province; “spec.” = specimens. Localities are given verbatim as appearing on the labels.

Xenopus (Silurana) mellotropicalis Evans, Carter, Greenbaum, Gvoždík, Kelley, McLaughlin, Pauwels, Portik, Stanley, Tinsley, Tobias & Blackburn, 2015: RBINS 13357–13358 (2 spec.), “Loango National Park, Ogooué-Maritime Prov., Gabon”.

Xenopus (Silurana) tropicalis (Gray): RBINS 165, “Bénué, Niger [*sic*; = probably Nigeria], Afrique occidentale”. RBINS 1811 (2 spec.), “Sénégal”. RBINS 13062 (2 spec.), “Tabou (4°25'N, 7°21'W), Ivory Coast”. RBINS 13028 (2 spec.) & RMCA 93-092-B-13–15 (3 spec.), “Lamin, western Gambia”. RMCA 93-092-B-24–27 (4 spec.), “Makumbaya, western Gambia”.

Xenopus (Xenopus) allofraseri Evans, Carter, Greenbaum, Gvoždík, Kelley, McLaughlin, Pauwels, Portik, Stanley, Tinsley, Tobias & Blackburn, 2015: RBINS 13359, “Loango National Park (2°20'37"S, 9°38'11"E), Ogooué-Maritime Prov., Gabon”. RBINS 13360, “Loango National Park (2°20'39"S, 9°38'12"E), Ogooué-Maritime Prov., Gabon”. RBINS 17089–17091 (3 spec.) & RBINS 17221, “Luki Reserve, Kongo Central, DRC”.

Xenopus (Xenopus) borealis Parker: RBINS 1094 (syntype), “Leikipia, 7–8000 feet, Kenya”.

Xenopus (Xenopus) gilli Rose & Hewitt: RBINS 7468, “Ottery, Cape Peninsula, South Africa”.

Xenopus (Xenopus) laevis (Daudin): RBINS 163, “Cap de Bonne Espérance” [= Cape of Good Hope, South Africa]. RBINS 163D, “Transvaal [= Transvaal Prov., South Africa]”.

Xenopus (Xenopus) muelleri (Peters): RBINS 9400 (9 spec.), “II/de/10, rivières à cours dénudé, Parc Nat. Garamba, Uele, Congo Belge [DRC]”.

Xenopus (Xenopus) poweri Hewitt, 1927: RBINS 4236 (7 spec.), “Mabwe, rive Est du lac Upemba, alt. 585 m, Parc Nat. de l’Upemba, Congo Belge [DRC]”.

Xenopus (Xenopus) parafraseri Evans, Carter, Greenbaum, Gvoždík, Kelley, McLaughlin, Pauwels, Portik, Stanley, Tinsley, Tobias & Blackburn, 2015: RBINS 17958–17959, “Ngoulmendjim (0°25'2.4"N, 10°34'4.7"E), Komo Department, Estuaire Prov., Gabon”.

Xenopus (Xenopus) pygmaeus Loumont: RBINS 18006, “Mukonoka River, Tshopo Prov., DRC.” RBINS 18204, “Mombongo, bank of the Congo River, Tshopo Prov., DRC”.

Xenopus (Xenopus) vestitus Laurent: RBINS 1811 (7 paratypes), “Rumangabo, Kinyantuku, Parc. N. Virunga, République du Zaïre [DRC]”. RBINS 1812 (paratype), “Rumangabo, Kinyantuku, Parc N. Virunga, Républ. Zaïre [DRC]”. RBINS 1813 (12 paratypes), “région Rutshuru, alt. ca. 1200 m, ex Parc N. Virunga, secteur sud, Républ. Zaïre [DRC]”. RBINS 1814 (paratype), “Rivière Bukengeri, alt. ca. 1200 m, région Rutshuru, ex Parc N. Virunga, secteur sud, Républ. Zaïre [DRC]”.

Xenopus (Xenopus) victorianus Ahl, 1924: RBINS 12675 (4 spec.), “Secteur Sud Rutshuru, Parc Nat. Virunga, Zaïre [DRC]”. RBINS 16835–16837 (3 spec.) & RBINS 17871, “Parc national de Rusizi, secteur Palmeraie, Burundi”. RBINS 16907, “Parc national de Rusizi, secteur Delta, Burundi”.

Xenopus (Xenopus) wittei Tinsley, Kobel & Fischberg: RBINS 1906 (37 paratypes), “Kalondo, alt. 1750 m, Lac Ndalaga, Mokoto, Parc Nat. Albert, Kivu, Congo belge [DRC]”. RBINS 1907 (4 paratypes), “Lac Magera, alt. 2000 m, Kivu, Zaïre [DRC]”. RBINS 1908 (4 paratypes), “Rivière Bishakishaki, alt. 2100 m, Kamatembe, Kivu, Zaïre [DRC]”. RBINS 1909 (9 paratypes), “Kanyabayongo, alt. 1750 m, Kabasha, Kivu, Zaïre [DRC]”.
